# Supplementary material for: Sub-picosecond thermalization dynamics in condensation of strongly coupled lattice plasmons
Source: Nat Commun. 2020 Jun 19;11:3139. doi: 10.1038/s41467-020-16906-1 (PMC7305221; doi:10.1038/s41467-020-16906-1)
Supplement: Supplementary file 1 — Supplementary Information [file 41467_2020_16906_MOESM1_ESM.pdf]

Supplementary Information –
Sub-picosecond thermalization dynamics in condensation of strongly coupled lattice plasmons

Väkeväinen et al.

Supplementary Figures

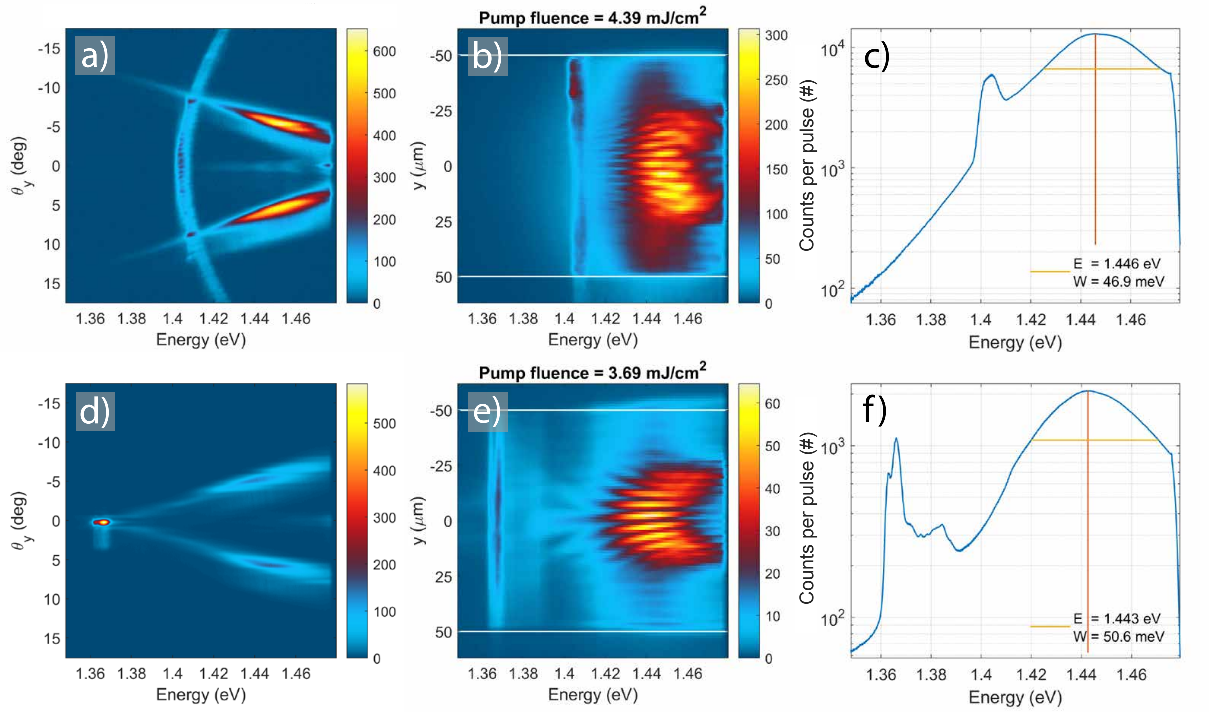

Supplementary Figure 1: **Effect of the band-edge energy.** Left column: k-space spectra in the TE mode direction. Middle column: Real space spectra. Right column: Line spectra obtained by integrating over the real space spectra in y-direction (between white lines). Results are shown for two lattice periods: (a-c) $p_y = 520$ nm and (d-f) $p_y = 590$ nm. Note that in (a) the curved band visible at 1.40...1.42 eV is the TM mode of the y-polarized nanoparticles ($p_x = 570$ nm).

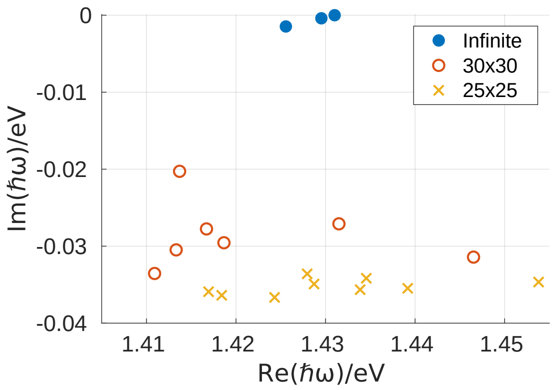

Supplementary Figure 2: **Results of the T-matrix simulation for infinite and finite arrays of nanoparticles.** Modes at $k = 0$ of infinite and finite rectangular arrays of cylindrical nanoparticles with periods $p_x = 621\,\mathrm{nm}$, $p_y = 571\,\mathrm{nm}$, radii $R = 50$ nm and height $h = 50$ nm, obtained by the multiple-scattering T-matrix method up to the octupole degree. Real and imaginary (representing loss) parts of the three distinct modes of an infinite lattice are shown by the blue dots. Mode energies (real and imaginary parts) for finite lattices of with $20{\times}20$ particles (yellow crosses) and $30{\times}30$ nanoparticles (red circles) show larger number of modes and also higher losses.

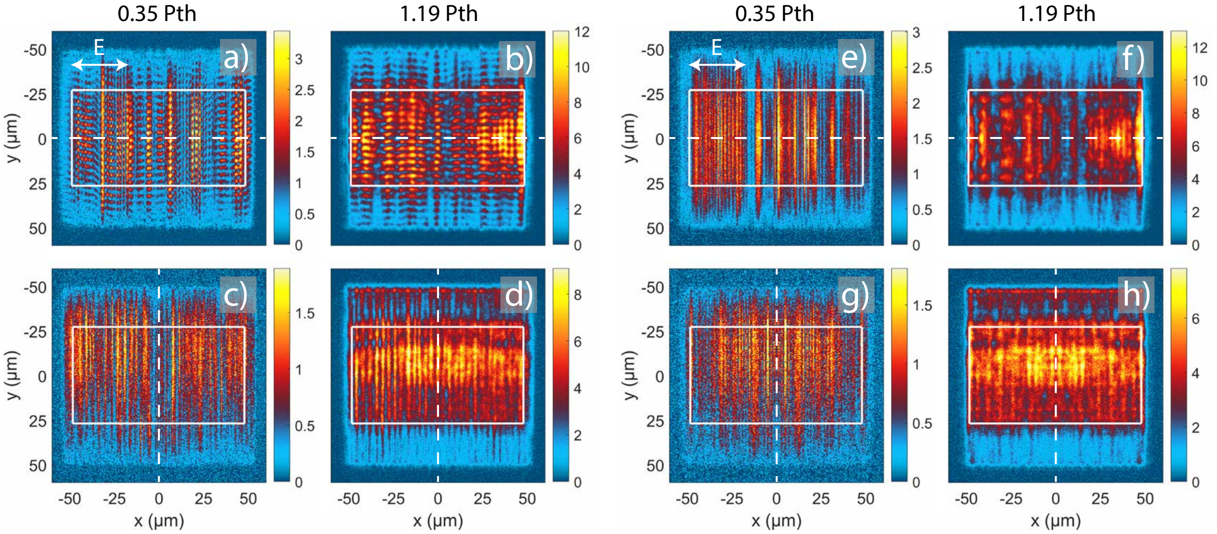

Supplementary Figure 3: **Spatial coherence measurement along y and x axis of the plasmonic lattice, with incoherently summed reference images.** (a-d) show the same images as the main text Figure 4, (e-h) show reference images for the same pump fluences. The reference images are obtained by inverting a real space image from one of the Michelson arms in the post processing, and summing it with the original non-inverted image. This is an incoherent equivalent of the coherently summed images in the Michelson interferometer. There is a clear difference between the interfered and non-interfered images in the y-direction coherence measurement (a,b,e,f) but not so clear in the x-direction measurement (c,d,g,h). For that, Fourier analysis of spatial frequencies is necessary to extracting the fringe contrast caused by spatial coherence. Here P_{th} means the threshold pump fluence for condensation and **E** refers to the pump polarization. The real space images are recorded for single pump pulses.

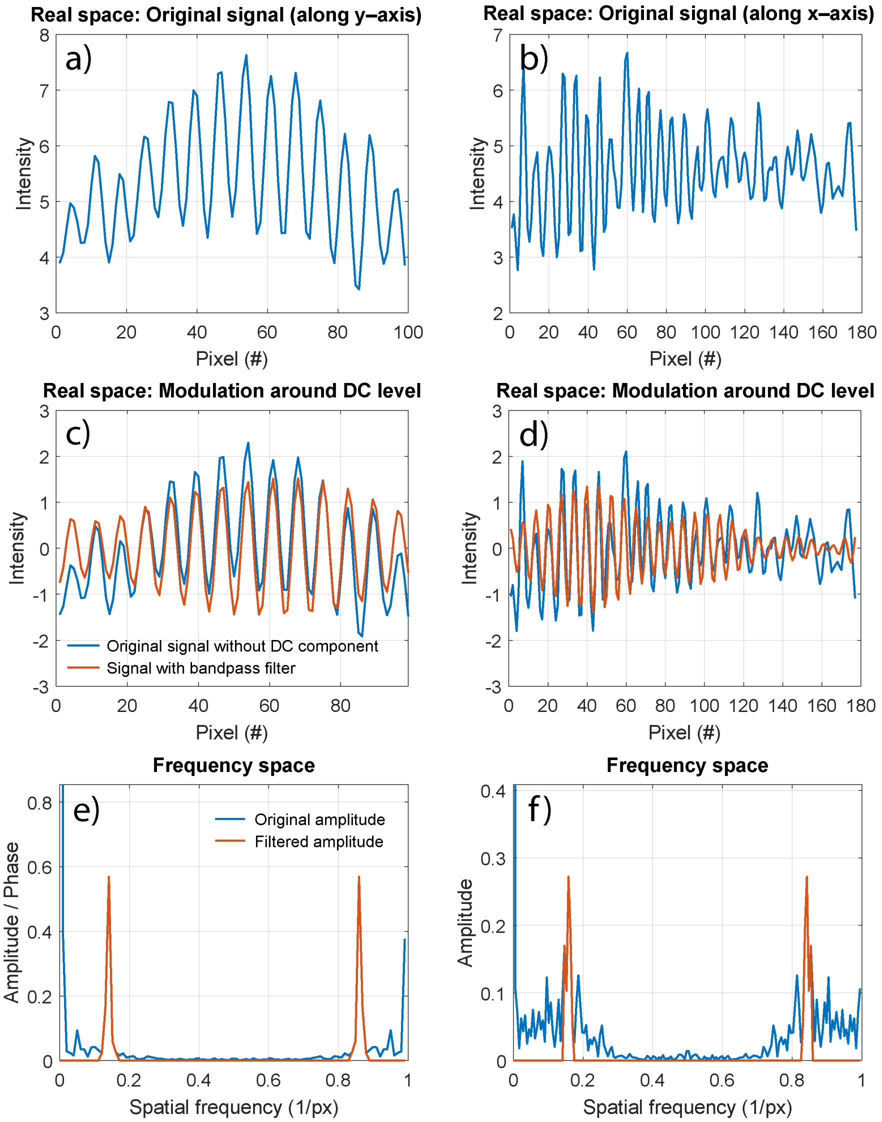

Supplementary Figure 4: **Fourier analysis method for extracting the fringe contrast in the Michelson interferometer images.** The left panel (a,c,e) shows the intensity along y-axis of the lattice, averaged over x (see white box in Supplementary Figure 3b), and the right panel (b,d,f) shows the intensity along x-axis of the lattice, averaged over y (Supplementary Figure 3d). In (c-f), the blue curves show the original signal and the red curves the filtered signal.

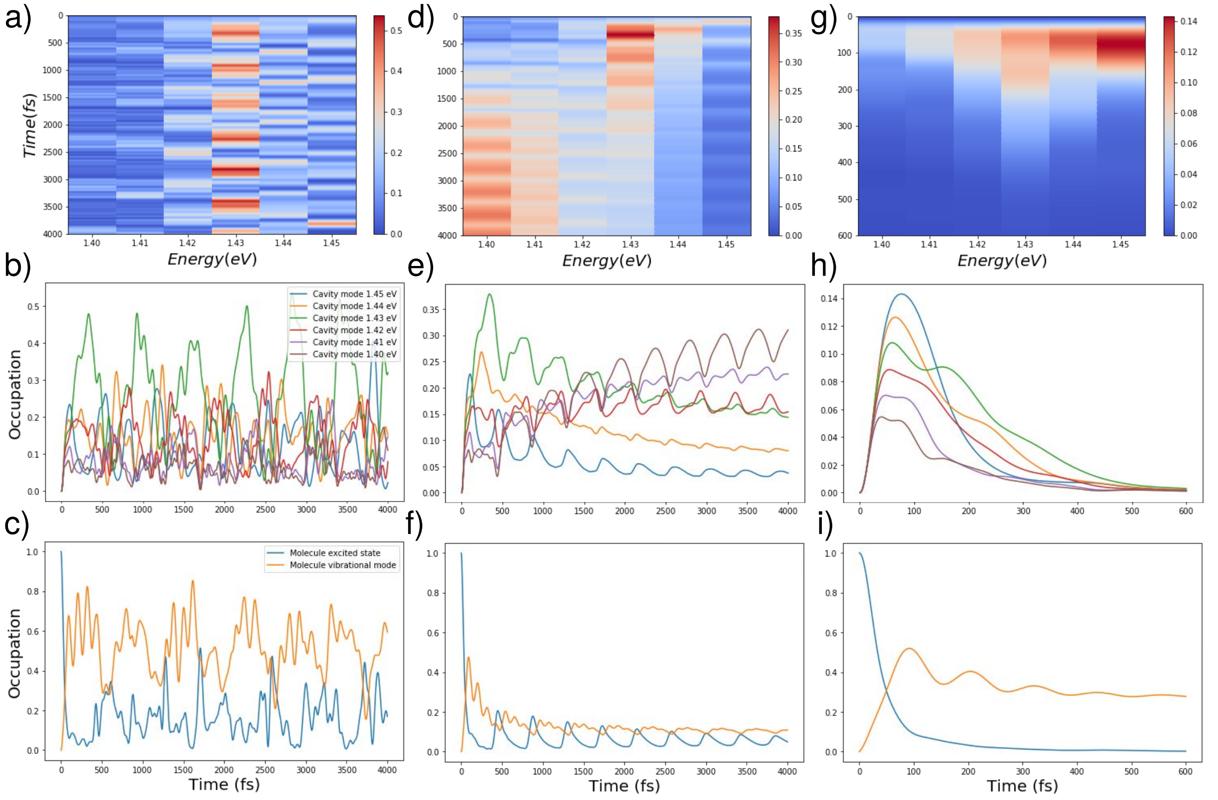

Supplementary Figure 5: **Results of the quantum model.** Time evolution of the occupation probability of cavity modes and the molecule excited state and the vibrational mode. Results are shown for the cases of (a-c) no losses, (d-f) only the vibrational mode loss, and (g-i) all losses present. The parameters used in the simulation are listed in Supplementary Note 6. Here (a,d,g) show the occupations of cavity modes at different energies in a color scale, and (b,e,h) their time evolution. Molecule electronic excited state and vibrational state population time evolution is shown in (c,f,i). When there are no losses (a-c), the occupation oscillates reversibly between different cavity modes and the molecule excited state. Applying the vibrational mode dissipation (d-f) results in red shift of the occupation towards the lowest-energy cavity mode. (g-i) shows that even in the presence of losses, the occupation reaches a lower-energy cavity mode before vanishing. Note that the cavity mode at 1.43 eV (green solid line in (b,e,h)) is favoured because it resides at the energy $\omega_\mathrm{m} - \omega_\mathrm{v} + \Delta$, in other words, it corresponds to a resonance where original excited state energy of the molecule is distributed to a vibrational excitation and the cavity mode. Here Δ is energy offset due to coupling between the molecule and the cavity modes. The shift can be approximated as $\Delta \approx \sum_i |g|^2/(\omega_\mathrm{m} - \omega_i)$.

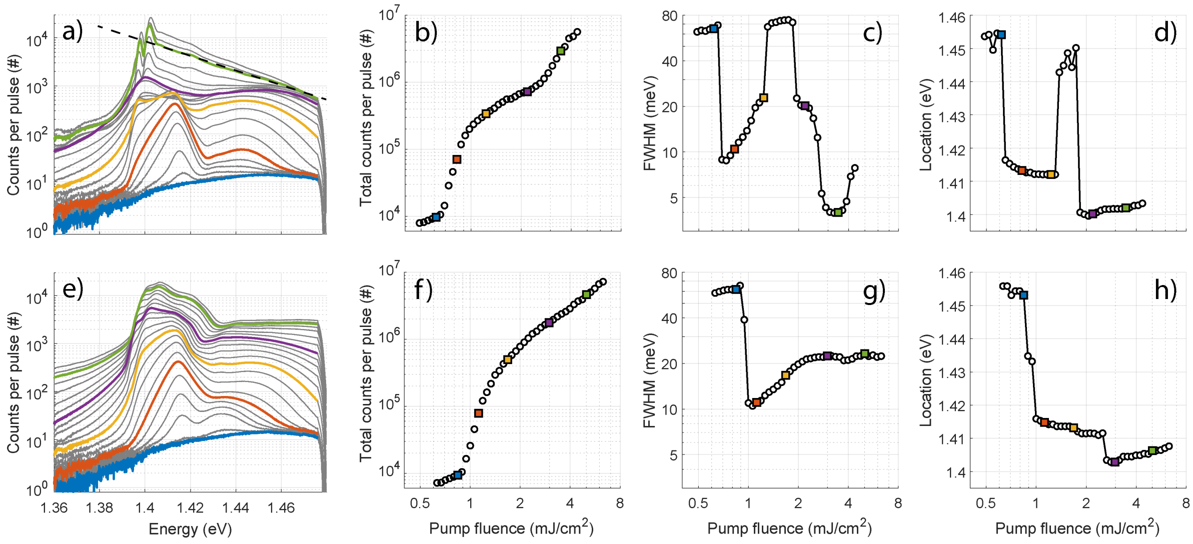

Supplementary Figure 6: **Pump fluence dependence and line spectra for 50 fs and 500 fs pulse durations.** First column: Population distribution at different pump fluences. Second column: Fluence dependence of the total luminescence, summed counts under the curves in (a,e), showing the threshold behaviour. Third column: The full-width at half-maximum (FWHM) of the spectral maximum as a function of pump fluence. Fourth column: The energy position of the spectral maximum. The results are shown for two excitation pulse durations: (a-d) 50 fs and (e-h) 500 fs. The pump fluences indicated by colored markers in (b-d,f-h) correspond to the colored lines in (a,e). The short excitation pulse results in the double threshold behaviour with distinct regimes of lasing, incomplete thermalization, and condensation, as explained in the main text. Before the onset of the first threshold (blue), the distribution reflects the spontaneous emission profile of the molecule. When the first threshold is reached (red), lasing peak is visible at around the band edge energy. After the first threshold (yellow and purple), population at broad range of higher energies is increased due to incomplete thermalization (intermediate regime). Condensation takes place at the second threshold (green), only for the 50 fs pulse, where narrow peaks are observed at the band edge, followed by a thermalized tail at the higher energies. Fit to the Maxwell–Boltzmann distribution (dashed line in (a)) gives the temperature of 313±2 K.

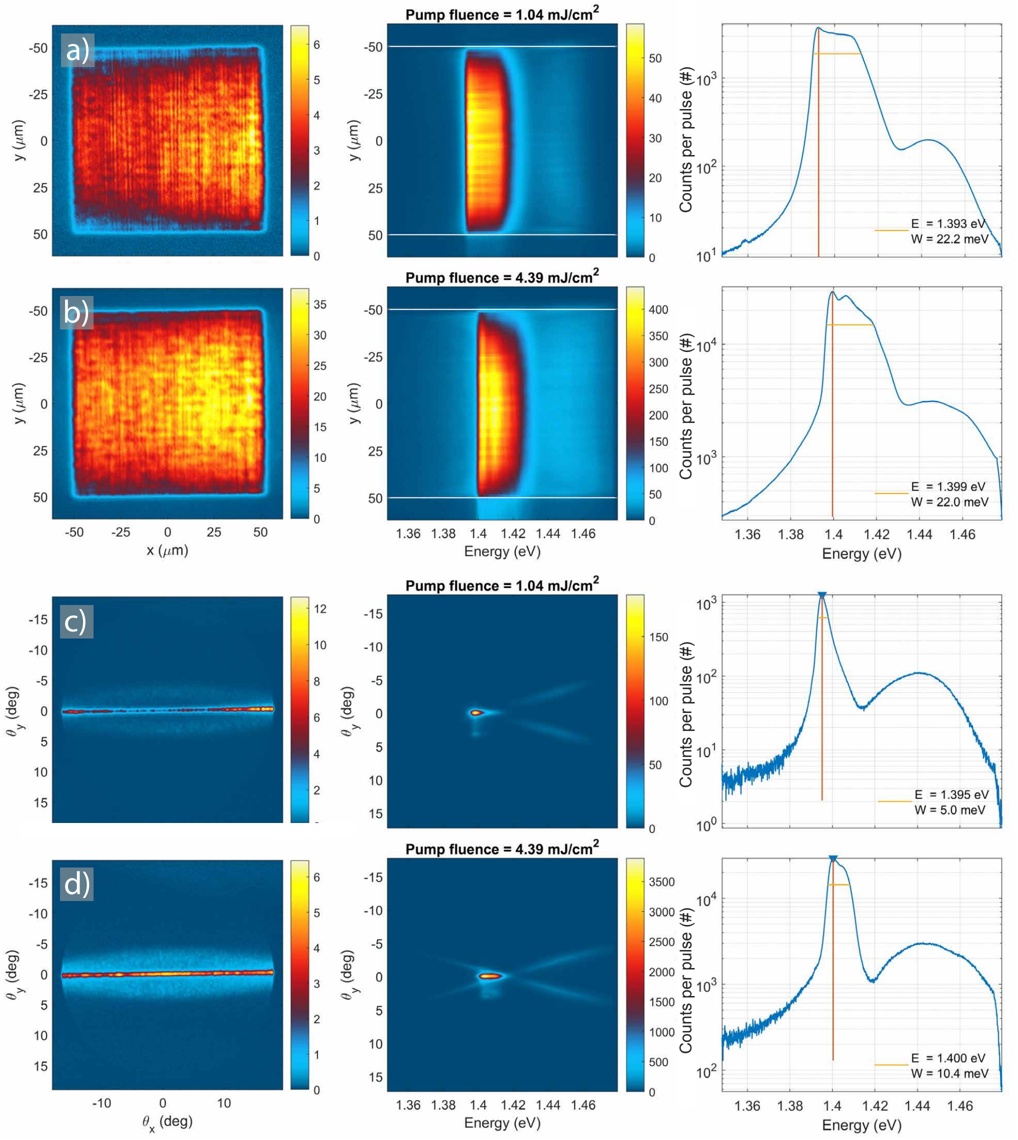

Supplementary Figure 7: **Real space and k-space measurement with 500 fs pulse duration.** (a-b) Real space measurements. Left column: Real space images. Middle column: Real space spectra. Right column: Line spectra of the intensity integrated over the real space spectra along y-axis between the white lines. (c-d) k-space measurements. Left column: 2D k-space images. Middle column: k-space spectra. Right column: Line spectra of the intensity integrated over the k-space spectra. The results are shown for a low (a,c) and a high (b,d) pump fluence. With a 500 fs pulse, we observe only the lasing regime where a narrow peak occurs at the band edge and some amplified spontaneous emission occurs in the dispersion branches, both above and below the crossing point. The luminescence from low to high pump fluences is spread widely in the TM mode, with little intensity seen at higher energies in the TE mode – no sign of thermalizing population nor 2D confinement. The results for the full range of pump fluences are presented in Supplementary Movie 3 (real space) and Supplementary Movie 4 (k-space).

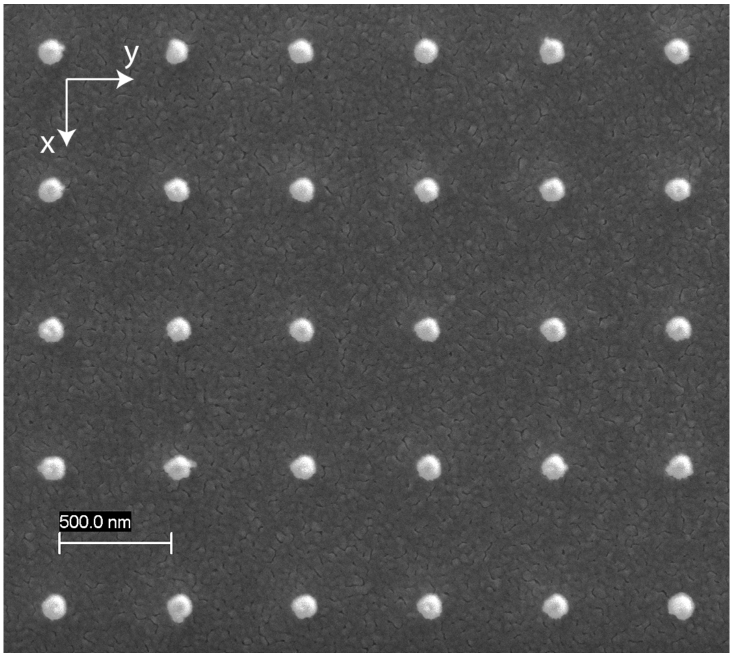

Supplementary Figure 8: **Zoomed-in scanning electron microscope image of a nanoparticle array.** The same image is shown as an inset in the main text Figure 1.

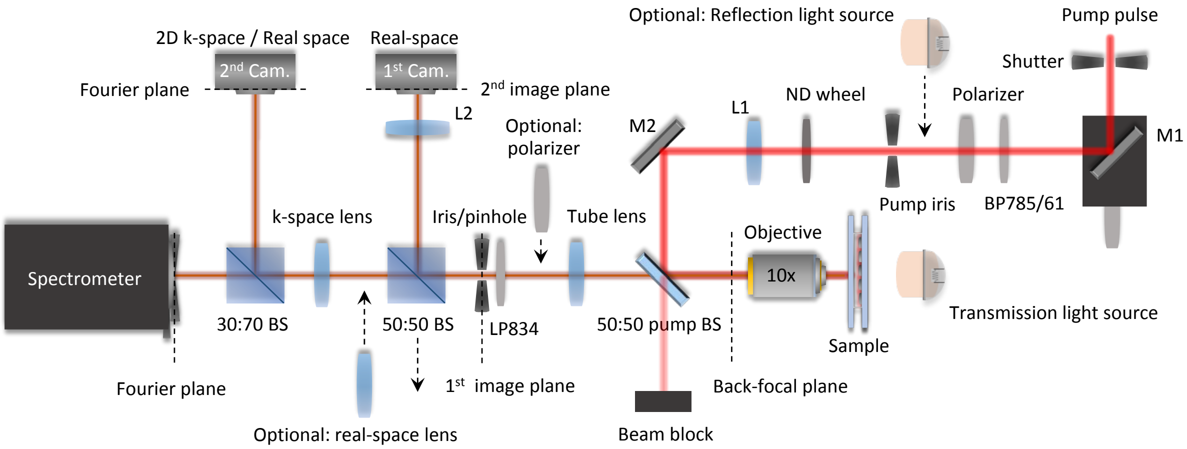

Supplementary Figure 9: **Schematic of the experimental setup.** The setup allows acquiring the real space, k-space, and spectral information simultaneously, and it is modular to measuring transmission, reflection, and the luminescence properties of the sample. Here, BP 785/61 refers to a band-pass filter with a bandwidth of 61 nm centered at 785 nm, LP 834 refers to a long-pass filter with a cutoff at 834 nm, M stands for mirror, L for lens, ND for neutral density, and BS stands for a beam splitter with a marked fraction of (R:T).

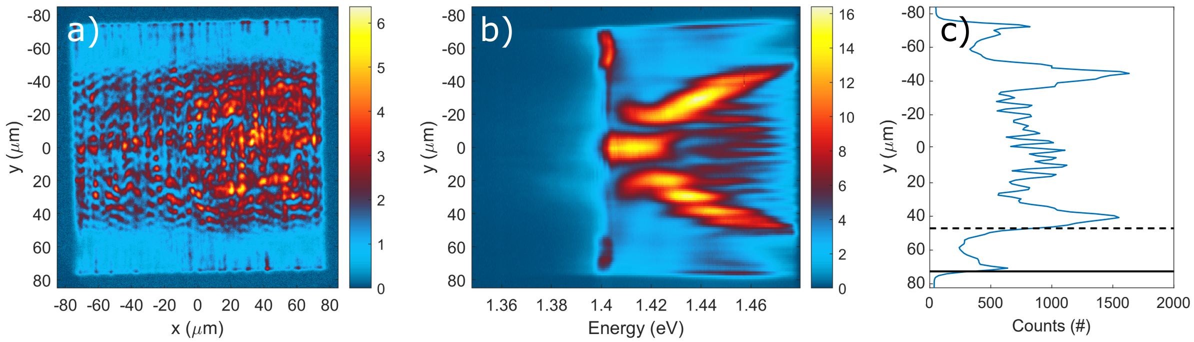

Supplementary Figure 10: **Definition of the location where red shift begins.** (a) Real space image, (b) real space spectrum, and (c) integrated intensity along the x-axis of the real space spectrum at intermediate pump fluence (2.2 mJcm^{-2}) for $150\times150\,\mu\text{m}^2$ lattice. Integration for the line spectrum is done over the high-energy range where the red shift begins (1.45$-$1.48 eV). We define the width of the dark zone as the distance between the array edge and the edge of the rising intensity in the real space image. This rising intensity edge corresponds to the location where the red shift begins in the real space spectrum (b). The array edge (solid line) and the edge of the rising intensity (i.e. starting point of red shift; dashed line) are defined at the half-maximum points of the rising intensity curve in (c). The distance between the solid and the dashed line is $25\,\mu\text{m}$.

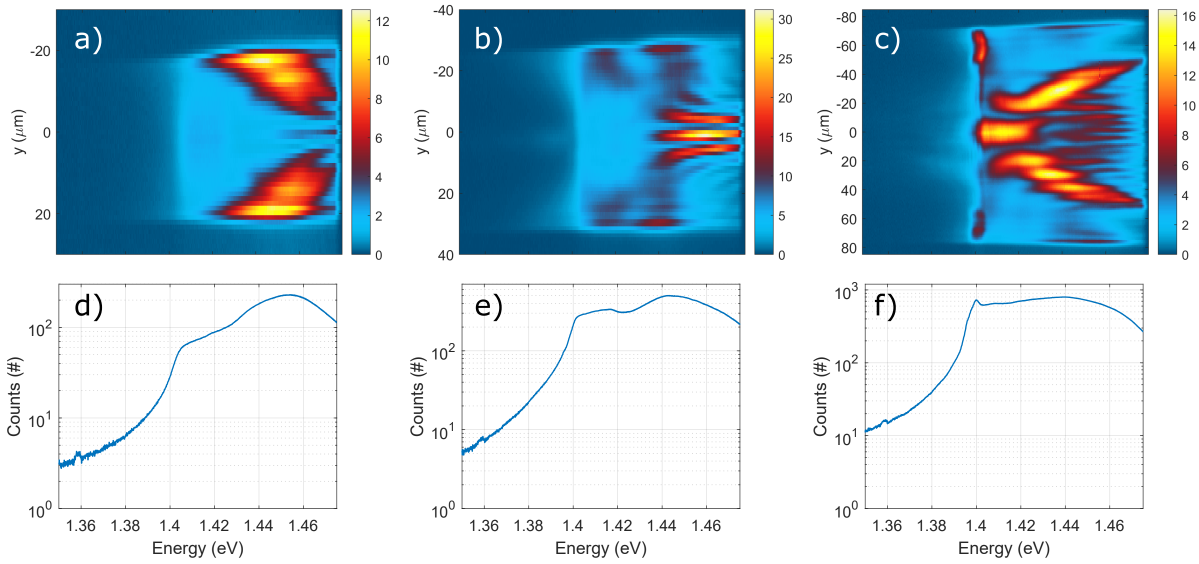

Supplementary Figure 11: **Line spectra for different lattice sizes.** Real space spectra (top row) and the corresponding line spectra (bottom row) at intermediate pump fluence (2.2 mJcm^{-2}) for lattice sizes (a,d) $40\times40\,\mu\text{m}^2$, (b,e) $60\times60\,\mu\text{m}^2$, and (c,f) $150\times150\,\mu\text{m}^2$.

Supplementary Tables

Supplementary Table 1: **Absorption and emission peak locations, maximum dissolving concentration, and notes on different dye molecules tested in lasing and condensation experiments.** If the absorption and emission wavelengths are measured, the used concentration (in the 1:2 DMSO:BA mixture) is given in parenthesis, otherwise a reference to the literature value is given. The column 'Max. c_{dye}' indicates the maximum concentration that has been successfully dissolved in the 1:2 DMSO:BA mixture. It may not be the highest possible value, but a note is given in the last column, if higher concentrations have been unsuccessfully tried. In addition, we have written down a mark how well the dye molecule has worked in lasing experiments (similar to the ones, e.g., in (*1–4*)). We have compared the quantum yield (QY) of the molecules at different concentrations, and found that for IR-140, the QY at 25 mM drops to 34% of the QY at 1 mM (highest efficiency). For IR-792 the QY at 25 mM drops to 6%, for IR-783 to 4%, and for IR-806 to 1.4%. At 100 mM, for IR-792 the QY drops to 1.4%, for IR-783 to 0.7%, and for IR-806 to 0.4%, respectively. IR-783 seems like a good candidate for the lasing experiments as its emission spectrum does not shift (or broaden) much when the concentration is increased, its QY stays decent as a function of concentration (>25 mM), and it emits something even at 280 mM (0.16% of the QY at 1 mM). Unfortunately, it bleaches quickly under optical excitation. All FEW dyes (*5*), particularly FEW S0260, appear as good candidates. However, IR-792 was chosen for this (and our previous (*6*)) studies as it was the first tested emitter that worked nicely in the lasing and condensation experiments at high concentrations.

	Abs. peak (nm)	Em. peak (nm)	Max. c_{dye}	Notes
Rhodamine 6G	530 (*7*)	552 (*7*)	100 mM	Good (at visible λ).
DCM	468 (*8*)	627 (*9*)	40 mM	Good (at visible λ).
IR-140 perchl.	835 (0.1 mM)	872 / 897 (1 / 25 mM)	25 mM	Good, insoluble at higher c_{dye}.
IR-792 perchl.	811 (150 mM)	845 / 858 (1 / 200 mM)	200 mM	Good.
IR-780 perchl.	780 (*8*)	834 / 844 (1 / 200 mM)	200 mM	Product discontinued.
IR-780 iodide	780 (*8*)	–	–	Dissolves very poorly.
IR-783	801 (0.1 mM)	829 / 840 (1 / 280 mM)	280 mM	Bad, bleaches quickly.
IR-806	827 (0.1 mM)	856 / 865 (1 / 100 mM)	100 mM	Bad, bleaches quickly.
IR-820	820 (*8*)	881 (100 mM)	100 mM	OK, not thoroughly tested.
FEW S0094	813 (*5*)	875 (100 mM)	100 mM	OK.
FEW S0260	816 (*5*)	867 / 877 (15 / 100 mM)	330 mM	Good.
FEW S0712	819 (*5*)	881 (100 mM)	200 mM	OK.
Styryl 9M	584 (*8*)	815 (*9*)	40 mM	OK, insoluble at higher c_{dye}.
Perylene Red	578 (*10*)	613 (*10*)	–	–

Supplementary Notes

Supplementary Note 1. Effect of the band-edge energy

We demonstrate the effect of the band edge location in Supplementary Figure 1 with two examples where the band edge is tuned either below or above the optimal energy of 1.40 eV. When the periodicity is set small so that the band edge of the x-polarized TE mode is at high energy (Supplementary Figure 1a-c), the propagation and red shift occurs along the lower dispersion branch of the TE mode, i.e., the red shift is not halted at the band edge. In contrast, when periodicity is set large so that the band edge resides at low energy (Supplementary Figure 1d-f), we observe the red shift of polaritons propagating along the upper dispersion branch of the TE mode but no condensation. The red-shifting population simply does not reach the band edge, which is at too low energy. The stripes in the real space spectra, Supplementary Figure 1b,e (also visible in main text Fig 2d,e and Figure 4c-f), arise from standing waves caused by counter-propagating modes. We found that the wavelength of the intensity oscillations is $\lambda_{\mathrm{RS}}(E) = \pi/k(E)$. Comparing the k-space and real space spectra shows in Supplementary Figure 1a-b that the oscillations are denser at lower energies because the lower energy corresponds to larger k. In contrast in Supplementary Figure 1d-e, the oscillations are sparser at lower energies because the lower energy corresponds to smaller k.

Interestingly while in previous studies using a dye molecule bath for photons (*11, 12*) or lattice plasmons (*6*) condensation required matching the lowest state energy with the energy where the molecule absorption vanishes, here that condition is not needed but the condensate formation is controlled by the interplay of the lattice size, periodicity and thermalization speed.

Supplementary Note 2. High luminescence signal of the condensate

As mentioned in the main text, we obtain roughly 5 orders of magnitude stronger signal compared to the first BEC in a plasmonic lattice (*6*). The increase of the signal is attributed to stimulated processes involved in our present experiments, and differences in the sample as well as the pump and detection geometry. In the previous study (*6*), the pump spot overlapped only partially with the nanoparticle array, and the condensate was detected over a small part of a long

array. The excited molecules emitted photons to propagating SLR modes, and the propagation took place in part of the array where there were only ground state molecules. This means that most of the excitations were lost during the propagation. In the present work, we pump over the whole array and also collect the luminescence from the whole array. Since the pump spot covers the whole array, all the propagation of excitations takes place in an area where there are excited molecules. The increased amount of excitations leads to a stimulated thermalization and condensation process which makes the excitations couple out as light in a very short time instead of decaying through the loss channels of the system. Since stimulated processes are involved, the increased amount of excitations leads to an output emission that is enhanced in a nonlinear manner. Furthermore, the samples that we use in the present work are more persistent towards photobleaching due to a very thick layer of dye solution (see Methods for details), enabling using higher pump fluences than in the previous work.

Supplementary Note 3. T-matrix simulation of lattice modes

To unveil the origin of the multiple modes, we have performed multiple-scattering T-matrix simulations of infinite and finite arrays of cylindrical nanoparticles, with the periods in x and y directions as well as the nanoparticle dimensions corresponding to our system. In the T-matrix approach, the scattering properties of a single nanoparticle are first described in terms of vector spherical wave functions (VSWFs), giving the T-matrix of the particle at a given frequency. For an individual particle, the nontrivial elements of the T-matrix are given by solving the scattering problem of the single particle. Next, the interactions between nanoparticles at different positions are expressed in terms of translation operators between the VSWFs with different origins (*13*). In case of infinite arrays, applying the Bloch boundary conditions, the electromagnetic response of a periodic nanoparticle array can be described with a matrix equation of the form

$$(I - TW)\, a = T p_{\mathrm{ext}} \tag{1}$$

where $T = T(\omega)$ is the single particle T-matrix, $W(\omega, \mathbf{k})$ is a lattice sum of the VSWF translation operators, a is a vector containing the coefficients of VSWFs scattered from a nanoparticle, and p_{ext} is a vector of incoming VSWF coefficients, describing the external fields driving the array. Lattice modes are defined as the solutions of Supplementary Equation (1) with the right

hand side set to zero (physically this means the waves propagate without the need of external driving) and exist only for such $(\omega, \mathbf{k})$ pairs for which the matrix $(I - TW)$ is singular (giving the dispersion relation of the array), which is equivalent to some singular value of the matrix being equal to zero. For finite arrays, the procedure is similar, but with different boundary conditions, as explained in (*3, 14*). In practice, the particles are lossy, hence the frequency ω needs to be complex if the wave vector $\mathbf{k}$ is real. We also exploit the symmetries of the system and evaluate the equation (1) separately for each irreducible representation of the little group corresponding to a given $\mathbf{k}$ vector. This gives us additional a priori information about the multipole polarizations of the particles in different modes. For a more detailed description of the method, see the Supporting Information of Ref. (*15*), and Ref. (*14*).

The blue dots in Supplementary Figure 2 show the singular values of $(I - TW)$ for cylindrical nanoparticles in an infinite array. Three distinct singular values appear at the Γ-point ($\mathbf{k} = 0$), while a large number of possible modes remain degenerate. This splitting into three modes comes from the finite size and cylindrical shape of the nanoparticles; in the empty lattice case all the modes are degenerate and we have seen by simulations that they remain essentially degenerate for spherical nanoparticles with less than 50 nm radius. Supplementary Figure 2 shows both the real and imaginary parts of the three distinct energies.

In finite lattices, the discrete translational symmetry of the infinite lattice is broken and the degeneracies present in the infinite case are further lifted. This is evident from Supplementary Figure 2 where the real and imaginary parts of the mode energies are shown for two different finite lattice sizes. A much larger number of distinct energies appears. The number of the modes and their energy separations and loss rates depend on the size of the lattice in a complicated way. The finite lattice size does not produce any simple "particle in a box" type distribution of the modes, since the finite size interplays with the internal multipolar modes of the nanoparticles in a non-trivial manner. Note that the number of modes found by the method is limited by the area in the real and imaginary frequency space from which the eigenvalues are searched. For the simulations in Supplementary Figure 2, the origin and the axes (real and imaginary parts of the energy) of the contour from within the eigenvalues are searched were $(1.431$ eV$+0.00\,\mathrm{i})$ and $(0.050$ eV$, 0.050\,\mathrm{i})$, respectively.

In the simulations of the finite size system, the discrete translational symmetry of an infinite

system cannot be utilized and the required computational time is significant and increases with the system size. Therefore we are not able to simulate arrays of the same sizes as used in the experiments. However, based on the results of Supplementary Figure 2, one can make the qualitative conclusions that an infinite lattice has three distinct energies and finite size lattices further ones, with energy splittings that are sensitive to the lattice size and properties of the nanoparticles. The energy splittings obtained by the simulations are of the same order of magnitude as the distances between the sub-peaks in the condensate in Figs. 2a and 3j of the main text.

Supplementary Note 4. Michelson interferometer experiment and Fourier analysis

The fringe contrast in the interfered images is extracted with a Fourier analysis. First, we need to find the period of the interference fringes arising due to coherence of $E(\mathbf{y})$ and $E(-\mathbf{y})$, and set a Fourier filter for spatial frequencies accordingly. The fringe period and the corresponding spatial frequency is determined by the incoming angle of the interfered images in the experimental setup. After that, the image data is gone through column by column, inside the region of interest, and a Matlab inbuilt Fast-Fourier Transform algorithm is performed to each pixel column at a time. In the spatial frequency spectrum, we find the peak value inside a predefined frequency bandwidth and compare that to the noise floor. The peak value needs to be above a chosen threshold value. If the peak is above the threshold, the rms-sum of the frequency components within the predefined bandwidth is compared to the background level (DC value of the frequency spectrum). Finally the contrast at certain pump fluence is taken as the mean value of the contrast along the columns inside the region of interest. The threshold value for a peak-acceptance level is adjusted so that the mean contrast value found in the reference cases (incoherently summed real space image) stays below 5%. Supplementary Figure 4 shows an explanatory example where the method is not applied for each pixel column separately but the intensity along y-axis averaged over x (from Supplementary Figure 3b), and the intensity along x-axis averaged over y (from Supplementary Figure 3d).

The stripes and fringes visible in Supplementary Figure 3 originate from properties of the nanoparticle arrays as well as from the Michelson intereferometer experiment. Let us first dis-

cuss the stripes specific to nanoparticle arrays. In our case one-dimensional lasing originates from linearly polarized dipolar nanoparticles. The nanoparticles are polarized in x-direction and predominantly radiate in y-direction. The polarization direction in nanoparticle array lasing is typically determined by the polarization of the pump beam, likely due to its coupling to the single particle resonance of the nanoparticle (*3, 16*). The feedback is provided by counter-propagating optical modes in y-direction, and long-range radiative coupling in x-direction is not efficient. This produces one-dimensional vertical stripes in the real space images (see Supplementary Figure 3a,c) (these can be understood as individual (or just a few neighbouring) nanoparticle chains lasing independently). Due to the one directional coupling, one dimensional lasing shows high spatial coherence only in the direction of the feedback.

Horizontal stripes in Supplementary Figure 3a are interference fringes that arise from overlapping two real space images, one of which is flipped with respect to the x-axis, at the camera sensor in the Michelson interferometer setup. There horizontal stripes (fringes) occur on top of the vertical lasing stripes. In Supplementary Figure 3c, the flipping is done with respect to the y-axis and since there is almost no spatial coherence in the x-direction, no additional fringes are obtained (confirmed by the Fourier analysis on the amplitude of spatial frequencies explained above).

In the condensation regime, there is a more uniform intensity pattern visible in the central part of the array and the Michelson interferometer produces the interference fringes in both x- and y-direction (see Supplementary Figure 3b,d). This is in contrast to the lasing regime that shows the interference fringes only in the y-direction (Supplementary Figure 3a). Note that with increasing the size of the nanoparticles, multipolar excitation of individual nanoparticles (*3, 17*) becomes possible and can facilitate two dimensional spatial coherence since the particle with multipole excitation can efficiently radiate in the two directions of the lattice plane. However, the nanoparticles in our current experiment (d = 100 nm) are too small (with respect to the wavelength range of interest) to exhibit 2D coherence in the lasing regime.

Finally, it is important to note that the Michelson interference fringes occur with a fixed period determined by the experimental setup (incoming angle of the overlapping images), and therefore these fringes can be distinguished from any other stripes in the real space images (with different period).

Supplementary Note 5. Rate-equation simulation of a stimulated-emission pulse

We use a standard four-level model for the gain medium to simulate the stimulated emission pulse when the four-level system is originally in its ground state, and excited with a 50 fs pump pulse. The levels are labeled as follows: the pump excites the system from the level 0 to 3, there is a non-radiative decay from 3 to 2, and emission to the cavity mode is from 2 to 1. The model shows the same temporal evolution after the pump pulse as a gain-switched laser, or a Q-switched laser after the Q-switch is opened (*18*). The transition lifetimes used for the four-level gain medium are: τ_{32} = τ_{10} = 50 fs and τ_{21} = τ_{20} = 500 ps, which are similar to those used in the literature for organic dye molecules (*1, 3, 4*). The spontaneous emission coupling factor (β–factor) is set to $\beta = 0.001$ and the cavity lifetime to $\tau_{\mathrm{cav}} = 100$ fs (corresponding to a typical lifetime of an SLR mode). The model is defined with the following coupled rate-equations, as in Ref. (*4*):

$$\frac{dn_{\mathrm{ph}}}{dt} = \beta n_{\mathrm{ph}} \frac{(N_2 - N_1)}{\tau_{21}} + \beta \frac{N_2}{\tau_{21}} - \frac{n_{\mathrm{ph}}}{\tau_{\mathrm{cav}}} \tag{2}$$

$$\frac{dN_0}{dt} = -rN_0 + \frac{N_2}{\tau_{20}} + \frac{N_1}{\tau_{10}} \tag{3}$$

$$\frac{dN_3}{dt} = rN_0 - \frac{N_3}{\tau_{32}} \tag{4}$$

$$\frac{dN_2}{dt} = -\beta n_{\mathrm{ph}} \frac{(N_2 - N_1)}{\tau_{21}} - \frac{N_2}{\tau_{21}} - \frac{N_2}{\tau_{20}} + \frac{N_3}{\tau_{32}} \tag{5}$$

$$\frac{dN_1}{dt} = \beta n_{\mathrm{ph}} \frac{(N_2 - N_1)}{\tau_{21}} + \frac{N_2}{\tau_{21}} - \frac{N_1}{\tau_{10}}, \tag{6}$$

where the populations of each level are denoted with N_i and the transition lifetimes with τ_i. Here, n_{ph} is the photon number in the mode (in our case the number of polaritons). Parameter r is the pump rate proportional to the pump intensity that has a Gaussian temporal shape.

In the model, the threshold value for population inversion is defined by comparing the gain and loss terms for the photon number in Supplementary Equation (2). The optical gain must

overcome the loss, and at the threshold they are equal

$$\beta n_{\mathrm{ph}} \frac{(N_2 - N_1)}{\tau_{21}} = \frac{n_{\mathrm{ph}}}{\tau_{\mathrm{cav}}}. \tag{7}$$

With a definition of $N^* = N_2 - N_1$, the threshold value becomes

$$N^*_{\mathrm{th}} = \frac{\tau_{21}}{\beta \tau_{\mathrm{cav}}}. \tag{8}$$

Supplementary Note 6. Description of the dissipative quantum model

We have studied the thermalization mechanism qualitatively with a microscopic quantum model including multiple cavity modes coupled to a single two-level system that is coupled to a shifted harmonic oscillator that describes the rotational-vibrational degrees of freedom within a molecule. The results of the model are presented in Supplementary Figure 5.

The system is described by the Holstein-Tavis-Cummings model (*19–23*) with the Hamiltonian ($\hbar = 1$)

$$H = \sum_i \omega_i a_i^\dagger a_i + \frac{\omega_{\mathrm{m}}}{2}\sigma^z + \sum_i \left(g a_i \sigma^+ + g^* a_i^\dagger \sigma^- \right) + \omega_v b^\dagger b + \omega_{\mathrm{v}} \sqrt{S}(b^\dagger + b)\sigma^{\mathrm{z}}. \tag{9}$$

Here $a_i^\dagger$ is the bosonic creation operator of the cavity mode of index i, σ^{z} and $\sigma^{\pm}$ are the Pauli operators describing the two-level structure of the molecule and $b^\dagger$ is the bosonic creation operator corresponding to the vibrational mode of the molecule. Furthermore, ω_{i} is the energy of the cavity mode of index i, ω_{v} is the energy of the vibrational mode, ω_{m} is the energy of the two-level system, g is the coupling between cavity modes and the molecule, and S is the Huang-Rhys parameter. Rotating wave approximation has been used in the simulation, assuming that the coupling $|g|$ is significantly smaller than the molecule and cavity mode frequencies. This is true with the parameters used in the simulation: $\omega_i = 1.40, 1.41, 1.42, 1.43, 1.44, 1.45$, $\omega_{\mathrm{m}} = 1.45$, and $g = 0.0075$ (in eV). Other parameters in the Hamiltonian used in the simulation are: $\omega_{\mathrm{v}} = 0.03$ eV and $S = 0.1$.

Dissipations of an open quantum system are taken into account in the Lindblad formalism, which yields the master equation (*24*):

$$\begin{aligned} \frac{\delta \rho(t)}{\delta t} =& \frac{i}{\hbar}[\rho(t), H] + \sum_i \kappa_i \mathcal{L}[a_i] + \gamma_{\mathrm{m}} \mathcal{L}[\sigma^-] + \gamma_{\mathrm{z}} \mathcal{L}[\sigma^{\mathrm{z}}] \\ &+ \gamma_{\mathrm{v},+} \mathcal{L}[b^\dagger - \sqrt{S}\sigma^{\mathrm{z}}] + \gamma_{\mathrm{v},-} \mathcal{L}[b - \sqrt{S}\sigma^{\mathrm{z}}], \end{aligned} \tag{10}$$

where $\mathcal{L}[O] = O\rho O^\dagger - \frac{1}{2}O^\dagger O\rho - \frac{1}{2}\rho O^\dagger O$ is the Lindblad superoperator, κ is the cavity dissipation rate, γ_m and γ_z are the radiative dissipation and dephasing rates of the molecule, and $\gamma_{\mathrm{v},\pm}$ are the rates that describe thermal excitation $(+)$ and dissipation $(-)$ of the vibrational mode. These rates are $\gamma_{\mathrm{v},+} = \gamma_\mathrm{v} n_\mathrm{B}$ and $\gamma_{\mathrm{v},-} = \gamma_\mathrm{v}(n_\mathrm{B} + 1)$, where $n_\mathrm{B} = 1/[\exp(\omega_\mathrm{v}/k_\mathrm{B}T_\mathrm{mol} - 1)]$ is the occupation probability of the vibrational mode of energy ω_v at thermal equilibrium. Solving Supplementary Equation (10) for $< a_i^\dagger a_i >$, $< \sigma^z >$, and $< b^\dagger b >$ gives the time evolution of occupation of the cavity modes and the molecule as well as excitation of the vibrational mode. Parameters used in the simulation for Supplementary Figure 5 are $\gamma_\mathrm{v} = 4\times10^{-3}$, $\gamma_\mathrm{m} = 1\times10^{-6}$, $\gamma_\mathrm{z} = 1 \times 10^{-3}$, $\kappa = 6 \times 10^{-3}$, and $T_\mathrm{mol} = 25 \times 10^{-3}$ (in eV). The simulation was performed using Python 3 with QuTiP toolbox (*25*). We use this model to illustrate a possible mechanism for the observed thermalization process, however, quantitative comparison to our experiments is not meaningful due to the simplicity of the model.

Supplementary Note 7. Different pump pulse durations

We studied the condensation phenomenon as a function of pump pulse duration and found that thermalization and condensation happens only for sub-250 fs pump pulses. Comparison of 50 fs and 500 fs pump pulses is presented in Supplementary Figure 6. It is evident that the longer excitation pulse results in only one (lasing) threshold. The distributions at around the threshold (blue and red curves in Supplementary Figure 6a,e are similar with both pulse durations, but at the higher fluences (yellow, purple, and green curves) the distributions are very different. With a 500 fs pulse, the population does not reach a thermal Maxwell–Boltzmann distribution, and no narrow peaks appear at the band edge. Besides the luminescence intensity, the different threshold behaviour is clearly visible in the FWHM curves (Supplementary Figure 6c,g). The FWHM is significantly decreased with both pulse durations at the first (lasing) threshold but only in the 50-fs case, the FWHM is decreased even further at the second (condensation) threshold. Note that at intermediate pump fluences, the 50 fs pulse shows a sharp increase of the FWHM because the maximum of the line spectra is found at higher energies (see Supplementary Figure 6d) as the thermalizing population dominates the signal. Examples of real space and k-space images and spectra for the 500 fs are shown in Supplementary Figure 7, for pump

fluences corresponding to the (a,c) lasing threshold and (b,d) beyond the condensation threshold of the 50 fs pump pulse. Importantly the real space and k-space images and spectra look nearly identical for both pump fluences for the 500 fs pulse duration, confirming that there indeed is no second threshold where the condensation would take place.

Supplementary Note 8. Estimation of polariton-polariton interaction strength

As mentioned in the main text, lasing and condensation take place at higher energy than the band edge of the lower polariton branch. Since the whole dispersion blue shifts as a function of pump fluence, the blue shift may be associated to degradation of strong coupling rather than e.g. Coulombic interactions. However, such saturation-caused non-linearity can also be considered as effective polariton-polariton interaction (*26*).

To make a rough estimation of the strength of such interaction based on the observed blue shift, we use a mean-field approximation estimate for low polariton density (linear regime) (*27*). In this case the blue shift ΔE is linearly dependent on the polariton density n and the interaction constant g, $\Delta E = gn$. Putting in our experimentally observed values of ΔE $\sim$ 20 meV and 10^{17} polaritons/m^2 we obtain a value of $g \sim 0.2\,\mu\text{eV}\mu\text{m}^2$. This value can be converted into a dimensionless interaction strength $\tilde{g} = gm/\hbar^2$ which can be defined for a two-dimensional system (*28, 29*). The polariton mass is estimated as in our previous work (*6*) by fitting a parabola to the band edge of the lower polariton dispersion branch. Fitting to both TM and TE modes of the coupled system gives an estimate of the effective mass in range $10^{-37}...10^{-35}$ kg. Using these values and the g calculated above, the dimensionless interaction strength $\tilde{g} = gm/\hbar^2$ is of the order of $10^{-7}...10^{-5}$.

Supplementary References

1. Zhou, W. *et al.* Lasing action in strongly coupled plasmonic nanocavity arrays. *Nature Nanotechnology* **8**, 506–511 (2013).

2. Yang, A. *et al.* Unidirectional Lasing from Template-Stripped Two-Dimensional Plasmonic Crystals. *ACS Nano* **9**, 11582–11588 (2015).

3. Hakala, T. K. *et al.* Lasing in dark and bright modes of a finite-sized plasmonic lattice. *Nature Communications* **8**, 13687 (2017).

4. Daskalakis, K. S., Väkeväinen, A. I., Martikainen, J.-P., Hakala, T. K. & Törmä, P. Ultrafast Pulse Generation in an Organic Nanoparticle-Array Laser. *Nano Letters* **18**, 2658–2665 (2018).

5. FEW-Chemicals. FEW Cyanine Dyes: 790-819 nm. https://www.few.de/en/menue-oben/spezialchemikalien/funktionelle-farbstoffe/loesungsmittelloesliche-cyanine/790-819-nm/. Accessed 2/6/2019.

6. Hakala, T. K. *et al.* Bose-Einstein condensation in a plasmonic lattice. *Nature Physics* **14**, 739–744 (2018).

7. OMLC. Rhodamine 6G absorption and emission spectra. https://omlc.org/spectra/PhotochemCAD/html/083.html. Accessed 2/6/2019.

8. Sigma-Aldrich. IR laser dyes, Rhodamine 6G, DCM, Styryl 9M. https://www.sigmaaldrich.com/finland.html. Accessed 2/6/2019.

9. Sirah-Lasertechnik. Laser Dyes 532 nm. http://www.sirah.com/dyes-accessories/laser-dyes-532-nm. Accessed 2/6/2019.

10. Kremer-Pigmente. Perylene Red. https://www.kremer-pigmente.com/de. Accessed 2/6/2019.

11. Klaers, J., Schmitt, J., Vewinger, F. & Weitz, M. Bose-Einstein condensation of photons in an optical microcavity. *Nature* **468**, 545–548 (2010).

12. Schmitt, J. *et al.* Thermalization kinetics of light: From laser dynamics to equilibrium condensation of photons. *Physical Review A* **92**, 011602 (2015).

13. Xu, Y.-l. Efficient Evaluation of Vector Translation Coefficients in Multiparticle Light-Scattering Theories. *Journal of Computational Physics* **139**, 137–165 (1998).

14. Nečada, M. & Törmä, P. T-matrix simulations in finite and infinite systems of electromagnetic scatterers. *In preparation* .

15. Guo, R., Nečada, M., Hakala, T. K., Väkeväinen, A. I. & Törmä, P. Lasing at K Points of a Honeycomb Plasmonic Lattice. *Physical Review Letters* **122**, 013901 (2019).

16. Wang, D. *et al.* Band-edge engineering for controlled multi-modal nanolasing in plasmonic superlattices. *Nature Nanotechnology* **12**, 889–894 (2017).

17. De Giorgi, M. *et al.* Interaction and Coherence of a Plasmon–Exciton Polariton Condensate. *ACS Photonics* **5**, 3666–3672 (2018).

18. Siegman, A. E. *Lasers* (University Science Books, Mill Valley, CA, 1986).

19. Tavis, M. & Cummings, F. W. Exact Solution for an N-Molecule–Radiation-Field Hamiltonian. *Physical Review* **170**, 379–384 (1968).

20. Ćwik, J. A., Reja, S., Littlewood, P. B. & Keeling, J. Polariton condensation with saturable molecules dressed by vibrational modes. *EPL (Europhysics Letters)* **105**, 47009 (2014).

21. Herrera, F. & Spano, F. C. Cavity-Controlled Chemistry in Molecular Ensembles. *Physical Review Letters* **116**, 238301 (2016).

22. Strashko, A., Kirton, P. & Keeling, J. Organic Polariton Lasing and the Weak to Strong Coupling Crossover. *Physical Review Letters* **121**, 193601 (2018).

23. Wu, N., Feist, J. & Garcia-Vidal, F. J. When polarons meet polaritons: Exciton-vibration interactions in organic molecules strongly coupled to confined light fields. *Physical Review B* **94**, 195409 (2016).

24. Breuer, H.-P. & Petruccione, F. *The Theory of Open Quantum Systems* (Oxford University Press, 2002).

25. Johansson, J. R., Nation, P. D. & Nori, F. QuTiP: An open-source Python framework for the dynamics of open quantum systems. *Computer Physics Communications* **183**, 1760–1772 (2012).

26. Carusotto, I. & Ciuti, C. Quantum fluids of light. *Reviews of Modern Physics* **85**, 299–366 (2013).

27. Deng, H., Haug, H. & Yamamoto, Y. Exciton-polariton Bose-Einstein condensation. *Reviews of Modern Physics* **82**, 1489–1537 (2010).

28. Bloch, I., Dalibard, J. & Zwerger, W. Many-body physics with ultracold gases. *Reviews of Modern Physics* **80**, 885–964 (2008).

29. Radonjić, M., Kopylov, W., Balaž, A. & Pelster, A. Interplay of coherent and dissipative dynamics in condensates of light. *New Journal of Physics* **20**, 055014 (2018).
