# Supplementary material for: Sub-picosecond thermalization dynamics in condensation of strongly coupled lattice plasmons
Source: Nat Commun. 2020 Jun 19;11:3139. doi: 10.1038/s41467-020-16906-1 (PMC7305221; doi:10.1038/s41467-020-16906-1)
Supplement: Supplementary file 3 — Description of Additional Supplementary Files [file 41467_2020_16906_MOESM3_ESM.pdf]

Description of Additional Supplementary Files

File name: Supplementary Movie 1

Description: Real space measurement for 50 fs pulse. Supplementary Movie 1 shows the real space measurement for the 50 fs pulse as a function of full range of pump fluences, corresponding to the experimental data presented in the main text Figure 2. Both the real space image and the real space spectrum are acquired simultaneously for each pump fluence. Such as in the main text Figure 2, the line spectra are obtained by integrating along *y*-axis between the white lines.

File name: Supplementary Movie 2

Description: *k*-space measurement for 50 fs pulse. Supplementary Movie 2 shows the *k*-space measurement for the 50 fs pulse as a function of full range of pump fluences, corresponding to the experimental data presented in the main text Figure 3. The 2D *k*-space image is acquired together with the TE mode crosscut (θ_x= 0; third figure from the left). The TM mode crosscut (θ_y = 0) is acquired separately by rotating the sample and the pump polarization by 90° (similarly to the *x*-direction spatial coherence measurement, see Methods). The white dashed line indicates the SLR dispersion without the dye molecules (main text Figure 1a), the white solid line indicates the lower part of the lower polariton branch (reflection maxima in the main text Figure 1d) and the black solid line indicates the upper part of the lower polariton branch (from the coupled modes model; main text Figure 1d). Such as in the main text Figure 3, the line spectra are obtained by integrating the TE crosscuts along k_y.

File name: Supplementary Movie 3

Description: Real space measurement for 500 fs pulse. Supplementary Movie 3 shows the real space measurement for the 500 fs pulse, corresponding to the experimental data presented in the main text Figure 6 and Supplementary Figure 7.

File name: Supplementary Movie 4

Description: *k*-space measurement for 500 fs pulse. Supplementary Movie 4 shows the *k*-space measurement for the 500 fs pulse, corresponding to the experimental data presented in the main text Figure 6 and Supplementary Figure 7.
